# Exploring the Unmet Needs of the Patients in the Outpatient Respiratory Medical Clinic: Patients versus Clinicians Perspectives

**DOI:** 10.1155/2015/749369

**Published:** 2015-12-09

**Authors:** Lone Birgitte Skov Jensen, Ulf Brinkjær, Kristian Larsen, Hanne Konradsen

**Affiliations:** ^1^Department of Education, University of Aarhus, 2400 Copenhagen, Denmark; ^2^Respiratory Department, Gentofte University Hospital, 2900 Gentofte, Denmark; ^3^Department of Education, Learning and Philosophy, University of Aalborg, 2450 Copenhagen, Denmark; ^4^Gentofte University Hospital, 2900 Gentofte, Denmark

## Abstract

*Aim*. Developing a theoretical framework explaining patients' behaviour and actions related to unmet needs during interactions with health care professionals in hospital-based outpatient respiratory medical clinics.* Background*. The outpatient respiratory medical clinic plays a prominent role in many patients' lives regarding treatment and counselling increasing the need for a better understanding of patients' perspective to the counselling of the health care professionals.* Design*. The study is exploratory and based on Charmaz's interpretation of grounded theory.* Methods*. The study included 65 field observations with a sample of 43 patients, 11 doctors, and 11 nurses, as well as 30 interviews with patients, conducted through theoretical sampling from three outpatient respiratory medical clinics in Denmark.* Findings*. The patients' efforts to share their significant stories triggered predominantly an adaptation or resistance behaviour, conceptualized as “fitting in” and “fighting back” behaviour, explaining the patients' counterreactions to unrecognized needs during the medical encounter.* Conclusion*. Firstly this study allows for a better understanding of patients' counterreactions in the time-pressured and, simultaneously, tight structured guidance program in the outpatient clinic. Secondly the study offers practical and ethical implications as to how health care professionals' attitudes towards patients can increase their ability to support emotional suffering and increase patient participation and responsiveness to guidance in the lifestyle changes.

## 1. Introduction

The burden of chronic respiratory diseases (CRDs) is increasing worldwide, and knowledge about the physical, mental, and social consequences of these diseases is extensive [1–4]. Generally, hospital outpatient clinics play a prominent role in the Nordic countries' health care systems. In Denmark, the number of outpatient visits to somatic hospital departments increased by 34% from 4,917,000 in 2002 to 6,612,000 in 2009 [5]. The outpatient respiratory medical clinic (ORMC) receives referrals after the patients' discharge from medical wards or by their general practitioner for the purpose of processing diagnosis, rehabilitation, and counselling or drug treatment. Many patients with CRD suffering from severe or very severe CRD are followed up regularly in the ORMC, according to the “chronic care model” guidelines and recommendations [6]. Patients who suffer from CRD may be vulnerable in many ways: CRD is associated with increased risk of anxiety, insomnia, fatigue, anorexia, loneliness, guilt, isolation, and depression [7–13]. As CRD progresses, it is characterized by severe daily symptoms such as shortness of breath, cough, and increased mucus production [1]. Thus, these diseases have not only physical but also existential implications in terms of their existence-threatening progressive and, over time, terminal character [14–16]. At the same time, it is well documented that many patients suffering from CRD may have limited economic and educational resources and, over time, a reduced and limited network [17–19]. In light of the fact that CRDs are characterized by their chronic, progressive, and lethal nature, there is a growing awareness of the need for more palliative-directed treatment and care for these patients [20, 21]. As suggested in other studies, the outpatient clinics are subject to many temporal, structural, and substantial requirements that may impact what may be talked about and how long the conversation can last [22–24]. These factors may limit opportunities to recognize and listen to patients' experiences during interactions [25]. Several studies suggest that patients' stories may offer important knowledge of how patients overcome, adapt to, and understand life with CRD [10, 26].

Furthermore, these stories may reveal strong emotions and existential suffering in patients [27–29]. The aspects of limited time and the many demands on the content of the consultation seem to influence what the health care professional (HCP) can hope to accomplish during counselling [22]. Several studies point to external factors affecting the lack of patient involvement and HCPs' responsiveness to patients' existential and emotional suffering [30], including aspects such as increasing work pressure, numerous changes in the structure of tasks, implementation of new or changed administrative tasks, and increasing demands for documentation [25, 31]. Furthermore, the demands of patients regarding empathy and the recognition of their suffering may exhaust HCPs and cause burnout [25, 31]. At the same time, many studies suggest that HCPs have a large potential to be instrumental in facilitating patients' coping and self-care management and optimizing their quality of life or, conversely, damaging these processes [8, 21, 32, 33].

Over the last several decades, the former authoritative approach of the HCP has been replaced by an ideological, institutional, and also vocational desire to include chronically ill patients in their own care and treatment [34–36]. Today, being patient-oriented is immensely popular with health authorities in many Western countries, as it reflects a moral philosophical approach in which the patient is regarded as unique and the multidimensionality of the human experience of illness is recognized through a relation that builds on multiple understandings of the patient's situation [37, 38]. Studies show that this paradigm change is not always implemented and present during the patient-HCP interaction [19, 39]. The studies emphasized here, based primarily on hospitalized CRD patients, suggest that the interaction with patients may be difficult for multiple reasons. The assumption was, consistent with other studies in related areas [22], that several specific characteristics affect the outpatient interaction. These characteristics arise from the typically short-term and very structured content and time frame for each consultation, which is of relevance to the extent and the content of the interaction since the demarcated time for counselling and treatment may create limitations for the interaction and HCPs' ability to meet the needs of patients. Whether this is the case in the ORMC has not been addressed in other studies exploring how the short time sessions affect the interaction in similar or particular ways. There seems to be a lack of studies exploring the clinical encounter in the ORMC [23, 24, 40] and none exploring the processes of interaction between patients and HCPs. Targeted attention to the patient's perspectives, reactions to actions, and behaviour could contribute to increased insight into patient's concerns and expectations. This study aims to contribute with knowledge in this area concerning the needs and behaviour of the patients and patients' reactions to the actions of the HCP's during the short-term counselling in the ORMC.

## 2. The study

### 2.1. Aim

The aim of the study was to develop a theoretical framework explaining patients' behaviour and actions related to unmet needs during interactions with health care professionals in hospital-based outpatient respiratory medical clinics.

### 2.2. Participants

This study includes 65 field observations and 30 individual interviews with patients with a sample of 43 patients, 11 doctors, and 11 nurses, in total 22 HCPs, from three ORMCs in Denmark during a period of 18 months in 2012-2013. The patients all suffered from CRD and were aged 45 to 91 years. The patients' experiences with the ORMC varied and spanned from initial contact to four years' association with several annually recurring consultations. The first 13 patients, 7 women and 6 men, were included exclusively for purposeful sampling conducting the initial field observations during patient-HCP interactions in clinic 3. Nine patients suffered from COPD and 2 from fibrosis, and 3 did not have a definitive diagnosis at the inclusion time. The purpose of the initial field observations was to explore possible central interactional themes. Out of the subsequent 30 included patients, 17 had COPD exclusively, 4 had COPD and asthma, 3 had emphysema, 2 had fibrosis, and 3 had other interstitial lung diseases. Demographic data of the participating patients are shown in Table 1.

A total of 22 HCPs, 11 doctors and 11 nurses, were included from the same 3 ORMCs as the patients in the field observations. The number and the distribution of field observations on patient/health care professional interactions are shown in Table 2. The HCPs were included to the extent that they were communicating with participating patients during the field observations, as illustrated in Table 2.

### 2.3. The Outpatient Respiratory Medical Clinic

The nurses and doctors worked in separate premises and with different tasks related to the patients. Doctor-patient conversation lasted 15–20 minutes and typically referred to the status of the disease, the effect of medicine, and possible revision, referring to rehabilitation in the municipality or hospital for further investigation and assessment. Nurse-patient conversation lasted 15–45 minutes and covered disease status, lung function tests, MRC breathlessness scale, and body mass index, as well as guidance in lifestyle changes and advice and recommendations related to these subjects. All included clinics used a primary nurse and doctor system for all CRD patients, and nurses and doctors held separate individual consultations in the included clinics.

### 2.4. Data Collection

The patients were included and observed at different stages of the outpatient clinic course, including initial consultations, subsequent visits, diagnosis meetings, and conclusive meetings. The patients were included upon arrival to the outpatient clinic and invited to participate, thus giving the researcher permission to observe the interaction during the visit. The patients were followed during the waiting time before and in between the conversations, which often included conversations with a nurse and then a doctor and occasionally a follow-up conversation with the nurse concerning medication intake or a new appointment in the ORMC. The field observations lasted between 20 minutes and 2.5 hours. All patients were followed during their entire stay in the clinic. Before the patient left, a home visit or a phone interview was scheduled. The researcher's field observations were nonparticipatory in the ORMC during the counselling and interactions between the patients and HCPs. Between examinations and interviews, the researcher participated in conversations with the patients, brought coffee, and listened to stories about, for example, everyday life, individual coping with illness and disease, and the patient's individual experiences of interactions with HCPs. While listening and talking, the interviewer wrote down important points, ideas, and statements as memos [41]. These relaxed and unrecorded conversations gave important information about the patients' experiences regarding the ORMC, everyday life at home, and relations with close relatives. Insofar as it was possible for the patient, the follow-up interview was performed within a week of the visit to strengthen both the patient's and the researcher's ability to remember important topics for conversation or other areas that the researchers wanted to cover. Out of the 30 interviews, 19 took place in the patients' own homes and 11 interviews were performed by phone, in accordance with the patient's needs and preferences. The interviews lasted between 25 minutes and 3 hours. All interviews were tape-recorded and transcribed verbatim afterwards. The interview guide was initially loosely structured, exploring issues concerning the patient's perspectives, expectations, experiences and outcome of the interaction, view on health and illness, and the personal and unique challenges and joys of everyday life. The focus of data collection of the included HCPs was to understand and explain how the HCP actions during the interaction triggered counterreactions and actions in patients.

The included field observations of the HCPs had the purpose of exploring the actions of the HCPs to increase the understanding of what patients reacted to and how patients reacted and identifying the meaning and significance attributed to the interaction when the needs of the patients were predominantly unmeet.

### 2.5. Ethical Considerations

All interviews and field observations have been carried out with respect to the HCPs' and patients' experiences and actions. It has been a balancing act to be loyal to the participants' understanding of their actions and interpretations while being able to say something more and something different than the respondents could say. The project was carried out in accordance with ICN's code of ethics for nursing research [42] and approved by the Danish Data Protection Agency (j.nr. 2011-41-6670). Oral and written information about the study was given to the participants, including information on anonymity, informed consent, confidentiality, and the right to end participation at any time without stating any reason. All personal identifiers have been removed or disguised, and the participants are not identifiable and cannot be identified through the details of the stories.

### 2.6. Design and Data Analysis

The study is exploratory and based on Charmaz's social constructionist interpretation of grounded theory [43–45]. An important source of inspiration from symbolic interactionism is an approach to the research field in which social processes are regarded as not only structural but also situational and changeable over time [46]. In line with this way of thinking, this study's focus is on researching the meaning and action in specific situations as close to an inner perspective as possible, acknowledging that it is not possible to duplicate the participants' experiences [45]. The coding process led to identifying what was happening in the data, explaining the elements of the emergent theory generating categories that were made more and more abstract as data were gathered to refine the theory. During* initial coding*, fragments of data—words, lines, segments, and incidents—were closely studied for their analytic import. During* focused coding*, the most fruitful initial codes were selected and tested against extensive data to determine their analytical importance to categories in accordance with the constant comparative method in grounded theory studies. The process of data collection and initial and focused coding ended when the categories were saturated and when gathering fresh data no longer revealed new theoretical insights or new properties of the categories. During the data coding and analysis, the focus was directed towards the fact that many patients apparently felt a lack of HCPs' responsiveness to their problems and perspectives during interactions and was given limited possibilities to influence what the conversation could include, which was found to be a prominent frustration for patients in the follow-up interview.

### 2.7. Rigour

The criteria for validating the findings followed Charmaz's credibility, originality, resonance, and usefulness [45, 47]. A clear and rigorous working process, as described in the data analysis, assured credibility.* Originality* can be met by the fact that this study offers new knowledge regarding patients' responses to unmet needs during interactions in an underexposed area where knowledge is limited. To achieve* resonance*, the study must be relevant to the participants. Several of the participating patients pointed out the importance of developing better understanding and visibility of their needs in the ORMC. At the same time, the participating HCPs showed great interest in the study and recognized, among other things, the need of the patients to express feelings during their stories in the time-pressed ORMC, and they confirmed and recognized the experience of not having the time to examine patients' perspectives of life experiences with illness.* Usefulness* may be difficult to assess at this stage. Subsequent studies should examine, adjust, and adapt the findings to further shape the theory and identify possible practical implications for HCPs' practice.

## 3. Findings: Striving to Share the Significant Story

During the coding and analysis process, it became clear that the unmet needs of the patient triggered counterreactions initially conceptualized as resistance and adaptation behaviour.

Based on the data, a decision was made to pursue the findings of the unmet needs of patients based on patients' perspective. This does not mean that no positive and stimulating interactions were identified. But a research decision based on the data led us to unfold the patients' behaviour and experiences related to interactions in which they did not feel seen, heard, and recognized focusing on partially as well as predominantly failed interactions on the basis of the patients' comprehension. To investigate and compare processes related to patients' need to be seen and heard, subsequent patients were sampled to ensure a variation in patient experiences to unmet needs. Further investigation during data collection verified or modified this first impression and led to the sample included in this study as listed in Table 1.

**Figure 1:**
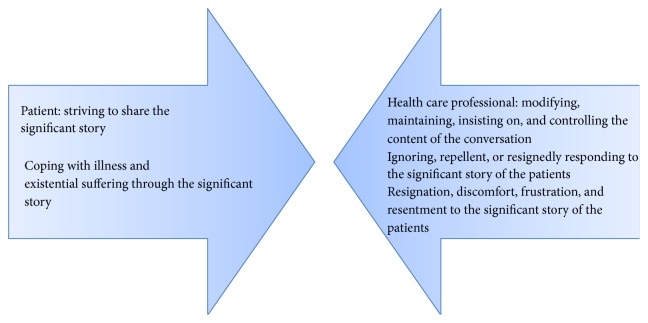
Lack of shared understanding to the content of the interaction in the outpatient respiratory medical clinic.

The analysis generated a substantive theory explaining how a main concern of many patients was “striving to share the significant story” comprising the significant individual issues related to their coping of illness or existential aspects of their everyday life. Regardless of the patients' behaviour, they rarely had the opportunity to tell their stories during the interactions. Patients' efforts to share their story and their reactions when this demand was unmet by the HCP triggered an adaptation or resistance behaviour, conceptualized as a “fitting in” or “fighting back” behaviour, explaining the patients' counterreactions when they were not seen, heard, and recognized during the medical encounter. The lack of shared understanding to the content of the interaction is illustrated in Figure 1.

**Figure 2:**
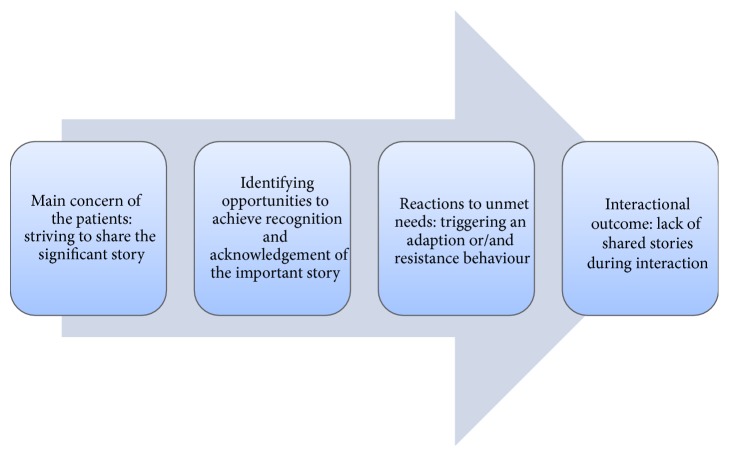
Core processes to unmet needs of the patients triggering counterreactions.

When the patients responded through “fitting in” behaviour, the HCP apparently assumed as if the patients were responsive and adaptive to the organized agenda. When patients responded through a “fighting back” behaviour, the HCP reacted with resignation, discomfort, frustration, or resentment. The “fitting in” and “fighting back” behaviour could overlap, balance, and shift during the interaction, and both behaviours could appear in the same interaction with varying strength at various times. Sometimes they would emerge during the interaction, and, in other situations, the patient had already chosen behaviour to the expected responses from the HCP. Neither of these two reactions created a basis for mutual understanding of the interaction, whether the patient adapted to a “fitting in” or “fighting back” behaviour. The counterreactions of the patients to HCPs' responses were affected by individual negotiation and adaptation in interactions regarding patients' efforts to play an active role during the interaction, their wanting of the HCP to respond to emotional statements, and their opportunities to modify their own expectations of content and counselling during the interaction. These recurring structural modes of patients were conceptualized as* role negotiation*,* emotional resonance*, and* perspective modification* modes and determinations of the meaning and significance attributed to the interactions when the needs of the patients were predominantly unmet. The process attributed to the interactions when the needs of the patients were predominantly unmet is explained in Figure 2.

The outcome of role negotiation, perspective modification, and emotional resonance in patients determined the behaviour of either “fitting in” or “fighting back” and whether the patient adapted to or resisted the HCP-planned guidance for procedures and issues during counselling.

The HCPs displayed several actions to modify, maintain, and control the content of the conversation. This includes HCPs' resignation to “let the patients talk” when they finished practical tasks. In other cases, the HCP could elude the patient's story by interruption, distraction, or an appeal to the fixed programme of the counselling session in order to win back control of the conversation. In other cases, the time for each session was used as an argument to prevent the patient from telling his or her story:

*We have to move on to… (HCP 14), if we are to finish all of this in time, we need to… (HCP 20), Let's stick with the lungs, shall we? (HCP 8)*

The various modification mechanisms used to minimize or exclude patients' stories included lack of reply, interruptions, listening in silence, lack of eye contact or physical contact, or referral to limited time. In some cases, the actions of the HCP resulted in the patient continuously insisting on telling the story, but mostly they closed down the patient's stories.

### 3.1. “Fitting In” Behaviour

“Fitting in” behaviour conceptualized a passive patient role, which maintained a mutually recognizable and, on the surface, smooth, evolving, and developing framework for interactions. “Fitting in” consisted of resignation, adjustment, and surrender behaviour during interactions by handing over control and the power to distinguish between important and less important topics of conversation. Patients gave up telling their significant story and adjusted to the HCP's objectives for conversation related to treatment or illness subjects concerning CRD recommendations, hospital standards, and guidelines. The HCP could reward “fitting in” behaviour with recognition of and attention to the patient's cooperation. “Fitting in” behaviour created a harmonic and consensual interaction concerning the content of the case and a mutual confirmation of the good atmosphere.

#### 3.1.1. Pretending Shared Understanding

Despite the fact that “fitting in” behaviour could be met with recognition and attention, this did not necessarily mean that patients were satisfied with the conversation or considered the interaction meaningful or relevant. On the other hand, “fitting in” behaviour often caused the HCP to assume the patient understood and recognized the importance and relevance of the counselling and treatment. The “fitting in” mode did not necessarily result in patients' receptiveness to counselling and information, as many patients silently resisted guidance while appearing responsive during the interaction. Below is an example showing a patient's “fitting in” behaviour and at the same time a resistance to counselling in the patient. During a field observation, the patient silently nods and does not argue with the HCP, who strongly disapproves of the patient's continued use of cough medicine. The patient explains in a subsequent interview:

*And I was thinking… “Don't talk to me that way,”… but then I thought, “No! Just finish your speech… I'm not listening anyway.” (Laughing out loud) (pt. 15)*

The “fitting in” behaviour resulted in patients rarely expressing their concerns, strategies, and emotions to the HCP. Several patients were preoccupied with symptoms of their other chronic diseases, which they found to be even more disabling, or troubled by a lack of understanding from relatives or feelings of loneliness or anxiety, which the interaction often rendered impossible to discuss. By contrast, the patient's questions could be context-related and in accordance with the HCP's predefined subjects, resulting in patient-directed and problem-based counselling.

### 3.2. “Fighting Back” Behaviour

“Fighting back” behaviour demonstrated resistance, retention, and frustration to the unmet needs of the patient. The role of the patient and of the HCP could be challenged or disturbed by the story. A patient's fighting back to create opportunities for the story without obtaining the HCP's responsiveness could furthermore create emotions such as anxiety, anger, despair, or resignation in the patient, as exemplified below:

*X (HCP), who we went to see, was very nice… and I think that she was very careful taking notes… So I got angry when we went to see Y (HCP)… Who does she think she is… talking about my back instead of talking about my lungs… I know how to treat my back… So that was not at all what I needed… And this commanding tone in her voice… “Do this and do that….” (pt. 15)*

Resignation could cause that patients gave up “fighting back” and reverted to “fitting in” behaviour, which was less energy demanding and made the interaction more predictable and less exhausting for them. The “fighting back” behaviour could relate to greater or lesser part of the interaction and consist of more or less important elements of patient's stories. “Fighting back” behaviour was often visible throughout the interaction. It was particularly evident in the many interactions where patients wanted to bring up other topics than planned or where patients considered the counselling dull, time-consuming, offensive, or invading.

*Well,… I had absolutely no impression that there was any contact between him and me. It was at the end, as he was becoming kind of offended and sulky. And my annoyance was particularly with the fact that I felt he started to become a little rude. However, it is non sequitur. You do not get any answers. In principle, I do not care whether it is negative or positive, but you must have some answers as to what the hell is wrong, has it become better or worse, what do we do and why not. And I do not think I got any answers at all. And as I said earlier… it's my big problem, or my big concern. It's my concern for people who cannot answer for themselves. So to me, the counselling, it's quite a waste of time. (pt. 24)*

In other cases, patients' “fighting back” behaviour seemed more veiled and expressed through a return to issues that the HCP had attempted to finish or through insistence on sharing a narrative or expressing their views on alternative treatment or the way they tackled common problems related to the illness. Many patients considered the bodily learned knowledge as important and tried to share these stories, even though the HCP rarely called for or responded to their experiences.

**Figure 3:**
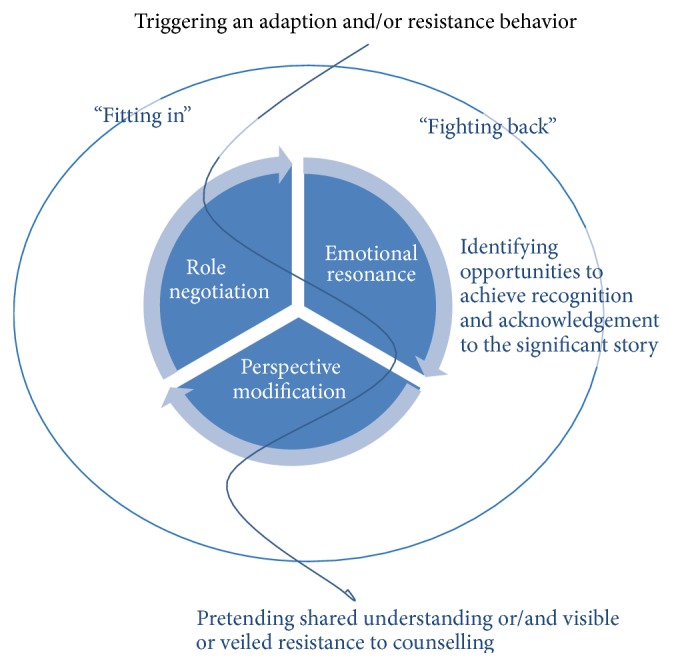
Striving to share the significant story. Triggering an adaption and/or resistance behaviour.

In Figure 3 the overall grounded theory is presented. This explains how “striving to share the significant story” triggered a “fitting in” or “fighting back” behaviour based on a process of* role negotiation*,* emotional resonance*, and* perspective modification* leading to the meaning and significance in patients attributed to the interactions when the needs of the patients were predominantly unmet. The wavy arrow-line down the centre of the figure illustrates how a “fitting in” as well as a “fighting back” behaviour in patients undergoes the same process of identifying perceived opportunity to achieve recognition through the process of role negotiation, emotional resonance, and perspective modification. The text below the figure explains how these processes in patients are leading to either a pretended shared understanding or a visible or veiled resistance to counselling (Figure 3).

### 3.3. Role Negotiation

Role negotiation describes the struggle for allocation of roles during the interaction and conceptualizes how the patient, silently or vocally, negotiates the patient role, including the right to define the content, the subject, and the framework for the interaction.

Patients could basically resist or adapt to the expected passive patient role the HCP expected them to undertake during interactions. At the same time, the HCP strived to uphold the dominant expert role and the right to define the content of the interaction, reacting with irritation, resignation, or frustration when challenged in the distribution of roles during the interaction. HCPs displayed several actions to accommodate this, including disregard, interruption, or breakdown of the story. Therefore, the patient could either fulfill or challenge the passive patient role. Role negotiation can be explained as patient-driven breakdowns, surrender, or attempt to control the content of the interaction in the ORMC.

#### 3.3.1. Breakdowns

When the interaction caused breakdowns, it was often clear to both parties. Many of the HCPs experienced these situations as unpleasant and tried to prevent or alleviate the disharmony caused by it. Breakdown situations clarified the patient's anger or frustration when the HCP's abortive assessment of the patient's emotional state became visible to both sides.

In one of the field observations, a dialogue between a patient and a nurse takes place illustrating the breakdown of the counselling. The patient suffers from posttraumatic stress syndrome, which, according to the patient, is a much greater challenge in his everyday life than his COPD. Early in the observed conversation, the HCP asks about the patient's daily number of cigarettes. The following dialogue takes place:

*(pt. 22): 15 cigarettes… Since August… only 15… I actually enjoy smoking.*

*HCP.1: How about electronic cigarettes?*

*(pt. 22): It's simply overrated… It's just bullshit… To quit smoking when you have PTSS (shakes his head).*

*HCP.1: How about nicotine patches?*

*(pt. 22): Yes, but I can't light them up, can I! (laughs) (field observation, pt. 22)*

The patient explained that he did not have the power to continue trying to make the HCP understand what his self-perceived biggest problems were: anxiety and water retention. In the follow-up interview, he pointed out that cigarettes were his only consolation and relief, the only thing that could alleviate his anxiety and ease his suffering. Even though patients mostly were aware that the HCP was not receptive to their story, it did not necessarily mean a change of behaviour. Patients' fighting back against the expected patient role rarely created opportunities for their story but instead complicated and disturbed the maintenance of the planned content at the ORMC. On the other hand, breaking or challenging the patient role created an opportunity to prevent the HCP from communicating information or recommendations to the patients they did not want to receive. This happened through irony, anger, humour, laughter, or bodily nonverbal rejection of the counselling. There was a connection between breaking or challenging the traditional patient role and the patient's experience of anger directed at the HCP and, at the same time, an experience of the ORMC as a place where the patient's problems were neither met nor recognized.

#### 3.3.2. Surrender

Patients could mitigate any previous challenge of the patient role by surrender through recognizing the HCP's advice or showing gratitude for the HCP's efforts to help them. When accepting the passive role, the patients surrendered and gave up expressing their wishes for the conversation and their needs to share and explore hope, emotions, and strategies. Instead, the patients followed the HCP's agenda during interaction and counselling. During the follow-up interview, the patients often expressed feelings of disappointment and dwindling expectations for the ORMC and the HCP.

#### 3.3.3. Control

Breakdowns often allowed the patient to take control of the interaction for a limited period of time. It rarely meant that the patient achieved breakthroughs in sharing perspectives and experiences. Regardless of the outcome of the “role distribution,” the patients' need and effort to share their significant story transformed their hope of creating opportunities during the interaction to a state of resignation, anger, or disappointment.

### 3.4. Perspective Modification

The patients' self-perception was often challenged and negotiated during interactions with the HCP in which patients tested their own attitudes and actions. Through this, patients were able to adjust to the situation and modify their own reactions to the actions of the HCP.

#### 3.4.1. Adjusting and Modifying

The HCP's violation, indifference, or recognition was thereby turned into something meaningful or meaningless by the patient. Through the story, patients modified and tested their own stances towards everyday life and the disease, strategies, self-perception, and the things perceived as important/not important for living with CRD. A consensus emerged between the HCP and the patient that it was the HCP's prerogative to comment on and reprimand the patient on issues such as weight, alcohol consumption, smoking, social network, moods, and strategies. The story of coping with CRD was important to many patients. These stories involved compromises on lifestyle changes that the HCP encouraged the patients to follow. The HCPs displayed inertia in their response to these compromises and a lack of involvement in the patients' own experiences of and tricks for coping with CRD. A pattern emerged in which the HCP often displayed an inflexible approach to counselling on lifestyle changes, regardless of the prerequisites, interest, or motivation of the patient. The HCP rarely responded to patients' own suggestions of strategies for living with breathlessness, which sometimes decreased patients' adjustment to the HCP counselling.

*Yes, I often see sulky attitudes at that outpatient clinic, actually… I mean, I don't think that they are very… enthusiastic… encouraging… making people want to pitch in… I think that they are a bunch of grumpy ladies… and I think that I wasted my time… by driving up here… I'd much rather go see XX, because he's sympathetic and seems enthusiastic about the things that you tell him… and what you accomplish on your own, right? (pt. 4)*

Some patients pointed to individual HCPs whom they considered to be particularly skilled and motivating in their effort to create lifestyle changes by adjusting and finding new ways and strategies. These HCPs displayed a recognizing approach to the patients' own actions in relation to lifestyle changes and reacted to the patients' emotional condition with advice, comfort, or encouragement.

### 3.5. Emotional Resonance

The presence or absence of emotional resonance was the key determinant of patients' self-perceived satisfaction or dissatisfaction to interactions and was characterized by patients' need to achieve emotional response and recognition during the interaction.

#### 3.5.1. Unrecognized Emotions

When the patients experienced a lack of emotional response to their story's emotional content, it resulted in additional suffering and discouragement in the patients. Many patients were striving to share some kind of hope expressed by the story and, at the same time, a need to have the emotions related to hope in the story recognized.

The HCP's rejection, ignoring, or lack of recognition of the emotional part of the story contributed to extinguishing the hope of less suffering, distress, and worries of everyday life. Many patients expressed the importance of emotional responsiveness during the interviews:

*I told her that I would like to quit the medicine… and she told me to forget about it,… but I told her that I had hoped,… but she wouldn't listen… And at that point in time, I just really needed the hope… I mean… that they understood that it was a hope of mine… or a milestone to me, right ? (pt. 4)*

The lack of emotional resonance negatively affected the patients' self-perceived motivation and courage to create everyday lifestyle changes. Furthermore, the HCPs' lack of emotional reflection on patients' challenges tended to increase the patients' “fighting back” mode to recommended lifestyle changes. The patients often reflected upon their own health and disease related choices and actions. Patients were aware that emotions such as triumph, pride, and satisfaction from reducing cigarettes or reducing medicine would not give rise to recognition by the HCP. Even if they wanted to share their enthusiasm for their own lifestyle changes, these stories were rarely considered good stories from the HCP's point of view. Apparently, there was a discrepancy between patients' and HCPs' experience of what a good patient story might imply. In some cases, the patients reacted to the discrepancy with anger, frustration, or fighting back towards the HCP. Other patients regarded the interaction as violating and did not feel encouraged to enter into a dialogue about lifestyle habits.

*No one bloody told me… “Damn, you managed to go from 30 cigarettes per day to 5, well done.” Nooo [sic]… “You need to quit smoking.” That's the way it is. (pt. 7)*

When the emotions in the story remained unrecognized, counselling sometimes became more anxiety provoking, incomprehensible, or less relevant, resulting in increased disappointment, despair, or hopelessness in patients.

#### 3.5.2. Emotional Recognition

When the HCP recognized and reflected the story about anxiety, despair, joy, victory, insecurity, resignation, or annoyance, it had a tremendous effect on patients. It was consistent across the data that patients responded positively and often with gratitude and appreciation when the HCP recognized and positively indicated an understanding of their difficult everyday life. It motivated them when the HCP recognized and really listened to the story and had great importance when they felt emotionally understood, when they were awarded a significant role, and when their effort to find strategies and perspectives was recognized. Some of the HCPs had the ability to reflect the patients' emotions by acting interested and engaged in their life. Comments like “It's not easy for you” and “I understand your plight” or leave-takings like “Keep up your spirit till we meet again” had great value for patients, who mentioned these HCPs as particularly talented, caring, and encouraging. The patients' stories of hope could be expressed by and converted into, for example, striving for more good days with less breathlessness, reducing the number of cigarettes, or using less medicine. It was consistent across data that, to the extent the patients were emotionally reflected, they found counselling to be more relevant and were more willing to explore the perspectives regarding the HCPs' attitudes to an optimal life with CRD.

## 4. Discussions

The original intention of this study was to explain interpretations and counterreactions to unmet needs in the patients during counselling with HCPs in hospital-based outpatient respiratory medical clinics. Patients' efforts to share their story and the subsequent rejection or ignoring of this pursuit triggered predominantly an adaptation or resistance behaviour, conceptualized as “fitting in” and “fighting back,” explaining the patients' counterreactions to unrecognized needs during the medical encounter, which turned out to be an exhausting but necessary behaviour to maintain perspectives and stories during the interactions. In addition, the patients' counterreactions were based on whether it was possible to play an active role during the interaction, the extent to which HCPs responded to the emotional intentions, and how the patients were able to change their own expectations of content during the interaction. These recurring structural patterns of interpretations of the patients, which were conceptualized as role negotiation, emotional resonance, and perspective modification conditions, turned out to be decisive of the meaning and significance patients attributed to the interaction and thus whether the patient adapted to a “fitting in” or “fighting back” behaviour. In accordance with the findings in this study, the patients' capability of being open to guidance was related to the extent to which they felt recognized and experienced the opportunity to share their concerns, suffering, and hopes with the HCP. The findings do not provide any answers to what seems to be a significantly greater degree of willingness to adhere to disease related issues and, to a lesser extent, an attachment to the emotional needs of patients. In addition, other studies have suggested that CRD patients lack knowledge of the disease and its long-term effects [48, 49] and that patients experience limited access to specialized nurses and doctors within the field of lung diseases [48, 50]. At the same time, it has been shown that lifestyle-related counselling and medicinal treatment at outpatient clinics can prevent readmittance, increase the level of patient satisfaction, and increase patients' ability to cope with illness and optimize drug compliance [15, 24, 51, 52]. However, before discussing the implication of these patterns of behaviour for clinical practice, it is essential to emphasize that although a general pattern of lacking commitment and responsiveness to patients' suffering in the ORMC was found, it may have complex causes and contexts, as suggested in other studies [25, 30]. Since this study maintains a patient-oriented perspective on the interaction, it is central to outline the limits and possibilities that affect the HCPs' actions in and attitudes to the interaction in the ORMC. Several studies suggest possible explanations for the lack of commitment to patients' emotional needs. Studies have shown that it is a difficult and complex task to maintain individual patient-oriented and emotionally responsive counselling, pointing to a high risk level of burnout and emotional exhaustion in many HCPs caused by the emotional needs, expectations, and demands of responsiveness from patients [25, 30, 31]. In terms of emotional burnout, lack of reciprocity, and attached concern, many studies suggest that the lack of responsiveness to patients' needs may be a way to resist emotional strain and stress [53, 54]. It may be a difficult task to listen to patients' stories of suffering, not knowing how to help [55]. Patients are often severely ill, treatment options poor, and healing possibilities nonexistent, and the existential suffering may be overwhelming to patients [1, 34]. At the same time, demands to navigate a time-constrained and topic-bounded guidance session involving many compulsory subjects to be documented may leave limited time for individual and patient defined selection of topics for conversation [22]. Additionally, HCPs are portrayed in a highly critical light in several studies, expressed particularly through the postmodern critique emerging from concepts like discipline, power, and individual control as problematic in the health care system [56–58]. The criticism that this study to some extent supports can give a misleading picture of the knowledge that HCPs possess and the practices associated with it. It needs to be taken into account that the HCPs included in this study had extensive knowledge and experience with CRD. Mostly they played a central role for patients in treatment and disease control, and they expressed a desire to support and help patients who suffered from CRD in the best possible way within the framework they had and the possibility they could recognize. At the same time, it is important to stress that the participating HCPs had extensive practical and theoretical knowledge regarding the consequences of the lifestyle choices made by patients suffering from CRD that they wish to introduce to patients, including knowledge on topics such as the benefit of exercise, smoking cessation, body weight, and medicine intake that may lead to extended life span and higher quality of everyday life in CRD patients [1, 34]. This means that there are many and often medically rational explanations to the HCPs' focus on control, monitoring, and treatment as found in this study. The vulnerability of patients suffering from CRD, which is prominent in our study and confirmed in other studies, stresses the importance of a supporting, empathic, and encouraging approach in HCP counselling [19, 39]. Although several other studies examine patient behaviour and actions [28, 39, 59], no other studies provide knowledge about the processes that constitute CRD patients' behaviour of fitting in or fighting back to counselling during interactions with HCPs in the ORMC. This study underlines the possibility of improving the experiences of being recognized in the ORMC through susceptibility of HCPs to listen to the stories of illness and suffering of the patients. The findings in this study suggest that an increasingly empathetic and extensive understanding of patients' concerns may possibly lead to a lesser degree of patients' resistance to advice and counselling during interactions. The findings suggest that patients rarely expected solutions to problems and concerns but rather anticipated an empathetic approach and willingness to listen to and understand their stories of suffering, hope, and ways of overcoming illness. This study offers new knowledge in an underexposed research field where the interaction is characterized by short meetings subject to a well-defined agenda during the clinical encounter. In accordance with other studies, our findings point to the importance of supplementing the disease-oriented perspective with a perspective increasingly inclusive of an empathetic and extensive understanding of patients' concerns and an appreciative view of patients [56, 60]. Further research is required to explore patients' counterreactions to unmet needs and further disclose the content and importance to patients with CRD in the ORMC.

## 5. Limitations

This study explores the patient's behaviour and actions related to interactions with HCPs in the ORMC. This study is limited by the fact that the voice of the HCPs is not explored in this study. Further exploration of the HCP's perspectives may provide further understanding of the knowledge of the ORMC interaction, which this study cannot provide. Future research should explore the HCP's reactions to the patient's actions and behaviour in the ORMC to provide an overall picture of reactions and counterreactions of both patients and HCPs.

## 6. Conclusion

The findings of this study allow for a better understanding of patients' counterreactions in the time-pressured and, simultaneously, tight structured guidance program in the ORMC. Firstly, the findings show that patients' efforts to share their story triggered predominantly an adaptation or resistance behaviour, conceptualized as “fitting in” and “fighting back,” explaining the patients' counterreactions to unrecognized needs during the medical encounter. Secondly, counterreactions of the patients were based on whether it was possible to play an active role during the interaction, the extent to which HCPs responded to the emotional intentions, and how the patients were able to change their own expectations of content during the interaction. The patients' capacity to receive help and guidance was related to the extent to which they felt recognized and experienced the opportunity to share their concerns, suffering, and hopes with the HCP. The study offers new knowledge in an underexposed research field where the interaction is characterized by short meetings subject to a well-defined agenda during the clinical encounter. The findings point to the importance of supplementing the disease-oriented perspective with a perspective increasingly inclusive of an empathetic and extensive understanding of patients' concerns and an appreciative view of patients. An increasingly empathetic understanding of patients' concerns may lead to less resistance in patients to HCP advice and counselling during interactions. Further research is required to explore patients' counterreactions to unmet needs and further disclose the content and importance to patients during counselling in the CRD.

## 7. Implications for Practice

The findings of patients' counterreactions to unmet needs provide new knowledge to HCP practice regarding the interactional possibilities and limitations in the ORMC. The findings offer practical and ethical implications as to how HCPs' attitudes towards patients can increase their ability to support emotional suffering and increase patient participation and guidance in the lifestyle changes that many HCPs hope to achieve during counselling.

Throughout this paper, how the lack of emotional responsiveness and recognition triggered a fitting in or fighting back behaviour has been outlined and analysed, explaining the patients' counterreactions to unmet needs during the interaction in the ORMC. This paper emphasizes a lack of shared expectations between the patient and HCP as being a central problem in the interaction, proposing that HCP may involve utilising the already available time frame to conduct an open dialogue with patients and prioritising the tasks and issues that are most important for the patient quality of life and health at the time. The need to develop specific models or guidelines, while focusing on the time-dependent ORMC interaction, is crucial in order to develop and strengthen an ORMC based on empathy, ethics, and emotional responsiveness to patients' needs and suffering during interaction in the ORMC. Future studies would benefit from examining how a narrative medicine could be implemented. A concrete proposal could be, for example, that future ORMC visits included an initial exploration of patients' experiences with the disease, hopes, ambitions, and expectations for the ORMC interaction. Furthermore, not only may a narrative approach be used in academic writing, but at the same time, it should be implemented as a way to forward the development of the ORMC. Actively using patient's stories is a way of understanding how the patient experiences can be understood and what is perceived as meaningful to patients regarding support, treatment, and guidance. Through this, the ORMC could gain development of the ORMC practice. Narrative methods seem to embrace the possibility of a more individual and patient-directed guidance, focusing on patient experienced problems rather than on the prospects based on the problems that HCPs perceive as typical, general, and specific to patient groups in the ORMC [39]. This approach would consequently be a shift in perspective and complement the HCP's understanding of the ORMC aims, tasks, and possibilities.

## Figures and Tables

**Table 1:** Demographic data, included patients.

Participant patients	Age	Clinic	Education	Employment	Lung disease	Stage	Chronic diseases
01–013 (7 female, 6 male)	—	3	—	—	6 COPD; 3 lung fibrosis; 3 without diagnosis	—	—
1 Female	68	1	White collar	Pensioner	Sarcoidosis	Mild	4
2 Male	52	1	Blue collar	Working	Alpha-1 antitrypsin deficiency	Severe	2
3 Male	59	1	Blue collar	Working		Moderate	2
4 Female	52	1	White collar	Working	Asthma; COPD	Moderate	2
5 Male	79	1	Blue collar	Pensioner	Emphysema	Mild	4
6 Female	45	1	Blue collar	Early retirement	COPD	Moderate	6
7 Male	49	1	Blue collar	Early retirement	Alpha-1 antitrypsin deficiency	Severe	1
8 Female	58	3	Academician	Working	COPD, asthma	Severe	2
9 Male	82	3	White collar	Pensioner	Emphysema	Moderate	2
10 Female	74	3	Employer	Pensioner	COPD	Moderate	2
11 Female	82	3	White collar	Pensioner	COPD	Moderate	3
12 Female	53	2	Blue collar	Working	COPD	Mild	2
13 Female	55	2	Blue collar	Sickness benefit	Asthma; COPD	Severe	2
14 Female	78	2	White collar	Pensioner	COPD	Moderate	2
15 Female	82	2	White collar	Pensioner	COPD	Moderate	2
16 Male	85	2	Blue collar	Pensioner	COPD	Moderate	1
17 Male	72	2	White collar	Pensioner	COPD	Severe	1
18 Female	91	2	White collar	Pensioner	COPD	Mild	2
19 Female	75	2	Academician	Pensioner	COPD	Moderate	1
20 Female	62	2	Academician	Early retirement	Emphysema	Severe	3
21 Female	66	3	Blue collar	Early retirement	COPD	Moderate	6
22 Male	52	3	White collar	Sickness benefit	COPD	Severe	3
23 Male	71	3	Blue collar	Pensioner	Pulmonary fibrosis	Severe	2
24 Male	59	2	Academician	Pensioner	COPD	Mild	2
25 Male	73	3	Blue collar	Pensioner	COPD	Moderate	2
26 Male	71	3	Blue collar	Pensioner	COPD	Moderate	1
27 Male	67	3	Blue collar	Pensioner	Pulmonary fibrosis	Severe	2
28 Male	62	3	Blue collar	Early retirement	COPD	Moderate	1
29 Female	82	3	White collar	Pensioner	COPD	Moderate	1
30 Female	58	2	Academician	Early retirement	Asthma; COPD	Severe	2

Stage: Spirometry measures airflow. Classification of patients' lung function is highlighted by the HCP during HCP-patient interaction and written down by researcher during the field observations.

**Table 2:** Field observations during patient-HCP interaction in ORMC.

HCP-nr.	Sex	Clinic	Profession	HCP counselling PT nr.
HCP 1	Female	3	RN	22; 29
HCP 2	Female	3	RN	26; 27
HCP 3	Female	3	RN	23; 28
HCP 4	Female	3	RN	2; 10
HCP 5	Female	3	RN	8; 9; 11; 24
HCP 6	Male	3	MD	9; 10; 23
HCP 7	Male	3	MD	24; 26; 27
HCP 8	Male	3	MD	01–013; 11; 22
HCP 9	Female	1	RN	2; 4
HCP 10	Female	1	RN	1; 3; 6
HCP 11	Female	1	MD	7
HCP 12	Male	1	MD	1; 3
HCP 13	Male	1	MD	5; 4; 6
HCP 14	Female	2	RN	16; 15; 17
HCP 15	Female	2	RN	20; 21; 30
HCP 16	Female	2	RN	13; 19
HCP 17	Female	2	RN	12; 14; 18
HCP 18	Male	2	MD	12; 13
HCP 19	Female	2	MD	21; 14
HCP 20	Female	2	MD	19; 25
HCP 21	Female	2	MD	15; 20
HCP 22	Male	2	MD	13; 14
Field observations in total:				**65**

HCP: healthcare professionals; RN: registered nurse; MD: medical doctor.
